# Assessment of Comorbidity in Patients with Drug-Resistant Tuberculosis

**DOI:** 10.3390/pathogens12121394

**Published:** 2023-11-27

**Authors:** Anna Starshinova, Michail Nazarenko, Ekaterina Belyaeva, Alexander Chuzhov, Nikolay Osipov, Dmitry Kudlay

**Affiliations:** 1 Almazov National Medical Research Centre, 197341 Saint-Petersburg, Russia; 2 Russia Pushkin TB Healthcare Dispensary, 196602 Pushkin, Russia; drpulmone@yandex.ru; 3 Scientific Research Institute of Phthisiopulmonology, 194064 Saint-Petersburg, Russia; 4 Republic TB Healthcare Dispensary, 185032 Petrozavodsk, Russia; ekaterina_83@bk.ru; 5 Interdistrict Petrograd-Primorsky TB Dispensary N. 3, 197343 Saint-Petersburg, Russia; chuzhov@mail.ru; 6 St. Petersburg State University, 199034 St. Petersburg, Russia; nicknick@pdmi.ras.ru; 7 Steklov Mathematical Institute of Russian Academy of Sciences, 191023 Saint-Petersburg, Russia; 8 Immunology Department, I.M. Sechenov First Moscow State Medical University, 197022 Moscow, Russia; d624254@gmail.com; 9 Institute of Immunology FMBA of Russia, 115478 Moscow, Russia

**Keywords:** comorbidities, multidrug-resistant tuberculosis, extensively drug-resistant tuberculosis, XDR, MDR, TB treatment success

## Abstract

A wide range of comorbidities, especially in multidrug-resistant tuberculosis (MDR-TB) and extensively drug-resistant tuberculosis (XDR-TB) patients, markedly complicates selecting effective treatment of tuberculosis (TB) and preventing the development of adverse events. At present, it is impossible to assess the severity of comorbid pathologies and develop indications for the administration of accompanying therapy in TB patients. The aim of this study was to identify the difference in the range of comorbidities between patients with MDR-TB and XDR-TB and assess the impact of comorbidities on TB treatment. Materials and Methods: A retrospective, prospective study was conducted where 307 patients with MDR-TB and XDR-TB pulmonary tuberculosis aged 18 to 75 years who received eTB treatment from 2016 to 2021 in St. Petersburg hospitals were analyzed. The analysis showed that the comorbidity level in MDR-TB and XDR-TB patients with TB treatment success and treatment failure was comparable with the use of the Charlson Comorbidity Index (CCI). The CCI demonstrated declining data in terms of TB treatment outcome period in both groups. A slight predominance of CCI score (3 to 4 points) in XDR-TB (22.7%) vs. MDR-TB (15.4%) patients was found. In the case of an TB treatment failure, the CCI level in MDR-TB vs. XDR-TB patients was characterized by a significantly higher rate of low magnitude (ranging from 1 to 2 points) in 21.1% vs. 4.5% (*p* < 0.05), which was higher in XDR-TB patients (ranging from 4 to 5 points, in 10.0% vs. 0, χ^2^ = 33.7 (*p* < 0.01)). Chronic viral hepatitis B and C infection, cardiovascular pathology, chronic obstructive pulmonary disease, and chronic alcoholism were found to be significant comorbidity factors that influenced the TB treatment success. Conclusions: It is evident that XDR-TB patients comprise a cohort with the most severe disease course due to comorbidities impacting TB treatment efficacy. The obtained data pointed to the need to determine comorbidity severity in patients with drug-resistant Mbt prior to administering TB treatment schemes.

## 1. Introduction

Since the onset of the COVID-19 pandemic, issues related to tuberculosis infections, including higher disease-related mortality and the detection of new tuberculosis cases, have become even more urgent [1]. Drug-resistant tuberculosis also continues to pose a threat to the global population due to an opportunity for the spreading of multidrug-resistant (MDR) and extensively drug-resistant (XDR) *M. tuberculosis* strains.

Low-efficacy treatment of TB is observed in multidrug-resistant tuberculosis (MDR-TB). According to the 2020–2021 WHO Report, MDR-TB treatment efficacy did not exceed 50–51% in the Russian Federation. This issue has not been solved currently [1,2]. At the same time, some studies have reported that the TB treatment efficacy of extensively drug-resistant tuberculosis is 40% of cases [3,4].

Many studies have shown that the presence of various concomitant pathologies, including HIV infection, has a significant impact on TB treatment efficacy and mortality from tuberculosis [4,5]. It was noted that a wide range of concomitant pathologies, as well as the presence of alcohol dependence and smoking, contributed to the formation of widespread drug resistance in the pathogen, which was associated with low adherence to therapy and withdrawal from TB treatment [6,7]. According to the available data, comorbid pathology of varying severity occurred in 68% of cases. It was shown that prechronic obstructive pulmonary disease (32%), chronic viral hepatitis (24%), and cardiovascular pathology (12%) were frequently present [8]. The presence of various concomitant pathologies led to the more frequent development of adverse reactions against the background of treatment, limitation to the prescription of the necessary combination of drugs, and the need to cancel TB treatment. Subsequently, all factors contributed to the growth of drug resistance of the pathogen, including resistance to new anti-TB drugs [5]. The use of new antituberculosis drugs could increase TB treatment effectiveness. Regrettably, the comorbidity status of TB patients and the high risk of drug-resistant *Mycobacterium tuberculosis* (Mtb) will lead to limiting the possibility of applying new anti-TB drugs in the future. It should be remembered that the development and introduction of new anti-TB drugs take tens of years.

The affected hepatobiliary system results in one of the most frequent antituberculosis therapy-related adverse events (AEs), especially following therapy involving MDR-TB and XDR-TB [8].

A wide range of comorbidities, especially in XDR-TB patients, markedly complicates the selection of proper effective TB treatment and the prevention of developing AEs [8,9].

Diabetes mellitus increases the risk of active TB and worsens its clinical course and outcome, and the growing burden of type 2 diabetes may support TB prevalence. At the same time, there is an opposite effect—TB affects glycemic control in people with diabetes due to inflammation and drug–drug interactions [10,11].

The effectiveness of TB treatment is mainly determined by the viability of the mycobacterial pool, including relevant drug resistance [12,13,14].

By affecting the viability of Mtb, drugs exert pathogen-specific effects in parallel with functional disorders in multiple patient systems [15,16,17].

Such adverse events often result from direct toxicity to healthy tissues due to the low specificity of cytostatic drugs or may be mediated via immune mechanisms. In addition, regardless of specificity, anticancer drugs may also exacerbate the course of patients’ comorbidities. The issue of chemotherapy-related toxicity is of paramount importance while treatment interventions proceed, thereby posing another therapeutic challenge [15,16].

At present, assessing the severity of comorbid pathologies and developing indications for the use of accompanying therapy in tuberculosis patients seem required. Moreover, no reliable data showing the impact of specific comorbid pathology on the effectiveness of TB treatment in the most severe disease courses, such as drug-resistant tuberculosis patient cohorts, have been obtained.

The aim of this study is to identify differences between the range of comorbidities in patients with multidrug-resistant (MDR-TB) and extensively drug-resistant (XDR-TB) tuberculosis as well as to assess the impact of comorbidities on the effective treatment of TB.

## 2. Methods

### 2.1. Patients

A retrospective and prospective study was carried out on 307 patients with MDR and XDR pulmonary tuberculosis, aged 18 to 75 years, who were treated in the years 2016 to 2021 in St. Petersburg hospitals, and the data obtained were analyzed. The inclusion of patients started in 2016. The assessment of the TB treatment effectiveness and long-term results, taking into account the influence of comorbidity, was possible in 228 TB patients by 2021. A comprehensive examination of patients was performed, assessing comorbidity status.

According to the study design, males and females with MDR and XDR pulmonary TB were analyzed; additionally, bacterial discharge was assessed and verified by bacteriologic methods in outcome period.

All patients met the inclusion and exclusion criteria. This study included patients who were treated with TB treatment, taking into account the data on the drug sensitivity of *M. tuberculosis* (Mbt) according to the results of the bacteriological conversion rates and the time to sputum smear and culture conversion Exclusion criteria were as follows: age under 18 years, pregnancy and breastfeeding, HIV infection (due to differences in comorbidity pathology, the need for antiviral therapy, and differences between patients with HIV infection in the level of immunosuppression), generalized tuberculosis, lack of chemotherapy, surgical treatment required at the time of enrollment into the study, and lack of patient’s consent to participate in the study. Patient characteristics are presented in Table 1.

**Figure 1:**
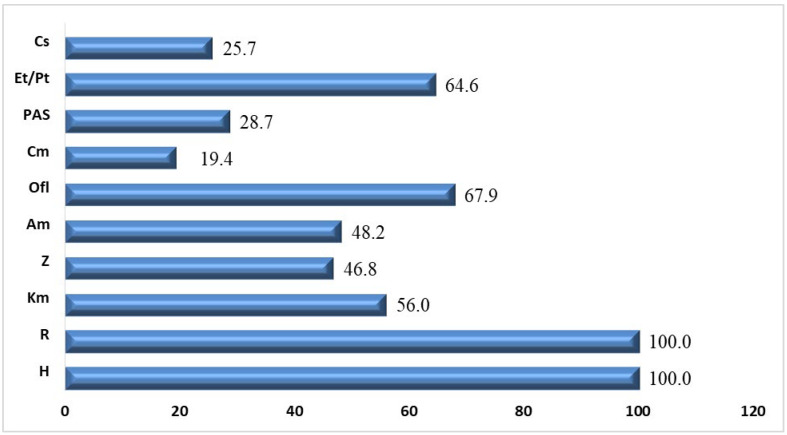
Total range of *M. tuberculosis* drug resistance based on bacteriological studies (%). Cs—cycloserine; Et/Pt—ethionamide/prothionamide; PAS—aminosalicylic acid; Cm—capreomycin; Ofl—fluoroquinolones; Am—amikacin; Z—pyrazinamide; Km—kanamycin; R—rifampicin; H—isoniazid.

The *M. tuberculosis* drug susceptibility range was assessed in all patients (Figure 1).

According to the data presented in Figure 1, *M. tuberculosis* resistance to aminoglycosides (kanamycin (Km), amikacin (Am)) and fluoroquinolones (Ofl) was detected most frequently, in 67.9% of cases, followed by thioamide derivatives (ethionamide (Et)/prothionamide (Pt)) and pyrazinamide found in 64.6% and 46.8% cases, respectively.

### 2.2. Methods

All patients underwent a comprehensive examination to further assess treatment success based on clinical, bacteriological excretion, and X-ray data. The treatment success (cured and treatment completed) with MDR-TB and XDR-TB patients was analyzed.

All patients with pulmonary tuberculosis received a therapeutic regimen taking into account the data on antituberculosis drug susceptibility, body weight, and concomitant pathology according to the current Russian Federation and international recommendations for MDR and XDR pulmonary TB treatment [18,19,20,21].

Antituberculosis drugs were selected based on antimycobacterial drug susceptibility data and consisted of combining five to eight drugs used for MDR-MBT and XDR-MBT treatment: ethambutol (E), pyrazinamide (Z), kanamycin (Km)/amikacin (Am) and polypeptide (capreomycin—Cm), fluoroquinolones (Fq), prothionamide (Pto), ethionamide (Eto), cycloserine (Cs)/terizidone (Trd), aminosalicylic acid (PAS), linezolid (Lzd), amoxicillin + clavulanic acid (Amx/Clv), imipenem (Imp)/cilastatin (Cln), meropenem (Mpm), bedaquiline (Bq), and thioureidoiminomethylpyridinium perchlorate (Tpp).

Currently, no scoring scales have been developed and used to assess comorbidity in tuberculosis patients. Here, the Charlson Comorbidity Index (1987), most often used in oncology, was applied [22]. In some tuberculosis studies, the Charlson Comorbidity Index (CCI) was used as a predictor of mortality [23] or for assessing the severity of diabetes mellitus in TB patients [24,25]. Currently, there are no indices or methods for assessing the severity of comorbid pathology in TB patients. The CCI, proposed for use in this study, allows for the inclusion of various system pathologies, as well as the presence of HIV infection, in the assessment. We did not find any other methods in the available literature, including the WHO recommendation. For this, a score scale was proposed by assigning a score level as follows: 1 point—myocardial infarction, heart failure, cerebrovascular disease, chronic non-specific lung disease, connective tissue disease, peptic ulcer disease, mild liver damage, diabetes mellitus; 2 points—hemiplegia, moderate or severe kidney damage, diabetes mellitus with damage to organs and systems, malignant neoplasms, leukemia or lymphoma; 3 points—moderate or severe liver damage; 6 points—oncopathology with metastases and HIV infection (AIDS stage); 1 point—every ten years of life in patients over 40.

### 2.3. Statistical Analysis

Statistical analysis of the data was performed using Statistica 10.0, SPSS 16.0 and GraphPad Prism 6 software. The sample size was sufficient to obtain statistically significant data. The mean parameter value (M ± m) was calculated and presented as M ± m, where M is arithmetic mean and m is mean error. The chi-square test (χ^2^) with the Yates correction was calculated, and the Mann–Whitney method for comparison of quantitative variables and the Student’s t and Fisher’s methods were applied. The risk ratio (relative risk (RR)), odds ratio (OR), confidence interval (95%CI), positive predictive value (PPV), and negative predictive value (NPV) were calculated using relevant formulas. OR and RR values greater than 1.0 were set as significant. Spearman’s rank correlation coefficient was used to evaluate interdependencies between the variables, where *p* ≤ 0.05 was set as significant. The Kaplan–Meier log-rank test and the Gehan–Breslow–Wilcoxon test were used to compare the overall and event-free survival rates between patient groups.

## 3. Results

To further assess the treatment success in outcome period using a multifactorial analysis for clinical, laboratory, and radiologic data, patients were divided into two groups based on bacteriologic examination data: Group I—MDR-TB (n = 157); Group II—XDR-TB (n = 150). A comorbidity-related impact on therapeutic effectiveness was also determined. A comparison of inter-group patient characteristics is presented in Table 2.

The data presented (Table 2) show that the length of therapy was longer in XDR-TB (II) than in MDR-TB (I) patients. Repeated TB treatment courses were applied three times more frequently in XDR-TB patients. Comorbidities were detected at a significantly higher rate in XDR-TB patients.

In stage 1, the TB treatment success in outcome period in patients with MDR and XDR pulmonary TB was analyzed (Table 3). According to the data presented (Table 3), the course of therapy was success at a significantly higher rate in MDR-TB patients than in XDR-TB patients (66.8% vs. 40.7%, χ^2^ = 20.6, *p* < 0.01), whereas lost to follow-up analysis was observed 2-fold more frequently in XDR-TB patients than in MDR-TB patients (16.7% vs. 3.8%, χ^2^ = 13.4, *p* < 0.01). Moreover, tuberculosis-related mortality rate reached 4.6% of XDR-TB cases (II).

**Figure 2:**
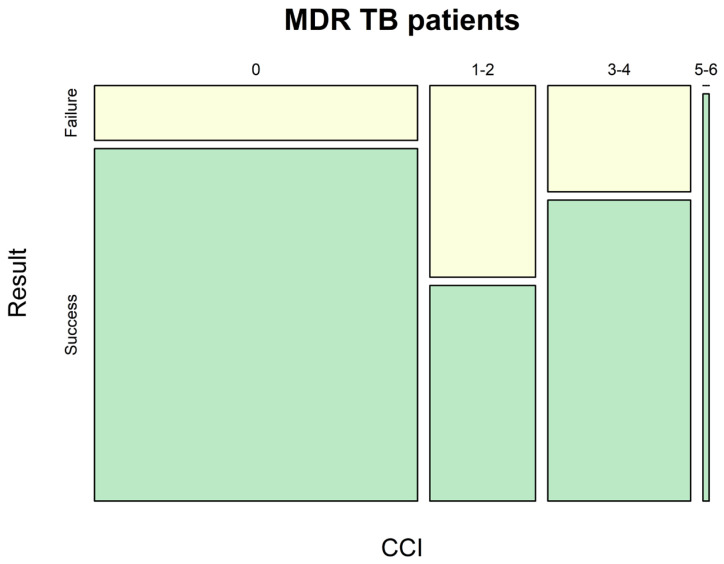
Impact of comorbidity on treatment success for patients with MDR-TB. CCI—Charlson Comorbidity Index.

It was found that XDR-TB patients (Group II) had significantly higher rates of viral hepatitis B and C that profoundly complicated the choice of therapy (Figure 2).

In the next stage, the CCI-based comorbidity score was assessed in 89 with MDR-TB and 139 with XDR-TB patients (Table 4).

It was demonstrated that severe comorbid status (score 3–4, 43.9% vs. 24.7%, *p* < 0.05; score 5–6, 10.7% vs. 0, *p* < 0.05) was significantly more frequent in XDR-TB patients than in MDR-TB patients (Table 4), whereas CCI level was significantly lower in pulmonary MDR-TB than in XDR-TB patients (41.6% vs. 20.9%, *p* < 0.05).

Further, according to the study design, we analyzed the level of comorbidity in the groups, taking into account treatment success and treatment failure for CCI scores higher than 1 (Table 5).

The analysis showed that the comorbidity level in MDR-TB and XDR-TB patients with TB treatment success and treatment failure was comparable with the use of the Charlson Comorbidity Index (CCI). The CCI demonstrated declining data in effective TB treatment course in both groups. A slight predominance of CCI score (3 to 4 points) was found in XDR-TB (22.7%) in comparison to MDR-TB (15.4%) patients.

In the case of an ineffective TB treatment course, the CCI level in MDR-TB patients vs. XDR-TB patients was characterized by a significantly higher rate of low magnitude (range of 1 to 2 points) in 21.1% vs. 4.5% (*p* < 0.05), which was higher in XDR-TB patients (range of 4 to 5 points, in 10.0% vs. 0, χ^2^ = 33.7 (*p* < 0.01)). Chronic viral hepatitis B and C infection, cardiovascular pathology, chronic obstructive pulmonary disease, and chronic alcoholism were recorded as significant comorbidity factors that influence treatment efficacy.

**Figure 3:**
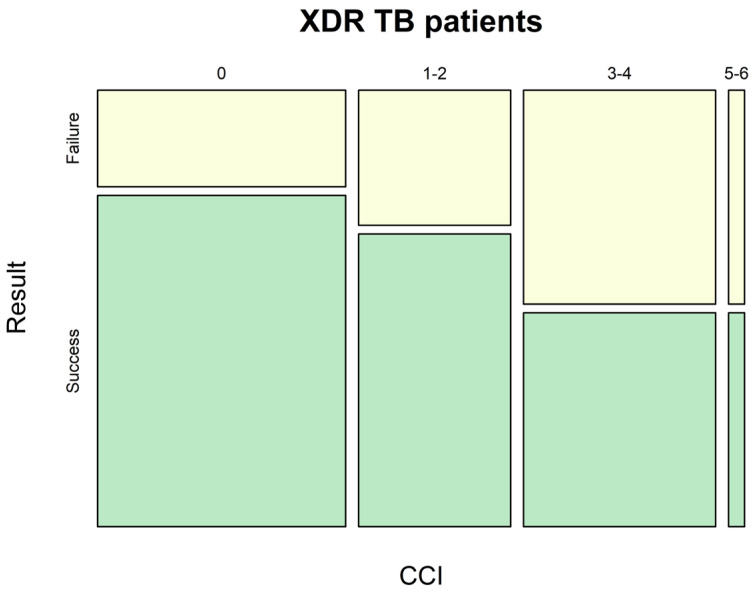
Impact of comorbidity on TB treatment success for patients with XDR-TB. CCI (Charlson Comorbidity Index).

An effect related to comorbidity level is shown in Figure 2 and Figure 3.

Among all patients with drug-resistant TB, CCI impacted solely the proportion of successfully treated MDR-TB and XDR-TB patients. Noteworthily, the treatment success tended to decrease while the comorbidity index became higher in XDR-TB patients (Figure 3).

The data were statistically analyzed to assess the significance level for the aforementioned tendency using the Cochran–Armitage trend test (Table 6).

The statistical analysis for inter-group CCI level, related to TB treatment success in MDR-TB patients, showed that it was not significantly affected by the magnitude of comorbidity status. At the same time, comorbid pathology in XDR-TB patients significantly more often impacted low TB treatment succuss (*p* = 0.0004, OR = 1.320, RR = 7.467), wherein it exerted an unfavorable effect based on positive and negative prognostic value (PPV = 0.9505, NPV = 0.2800).

In addition, 31.2% of XDR-TB patients with TB treatment failure had no comorbidities. Furthermore, we analyzed the data on concomitant pathology in 42% XDR-TB patients upon an treatment failure course (Table 7).

XDR-TB patients with treatment failure showed a virtually similar range of concomitant pathologies. Compared with other comorbidities, gastrointestinal tract comorbidities, particularly gastric and duodenal ulcer, chronic pancreatitis, and gastritis pathology, were found at a significantly higher rate. The majority of patients in the subgroups were represented by subjects with chronic viral hepatitis B and C infection, which may be accompanied by a high risk of hepatotoxic reactions. In isolated cases, cardiovascular pathology manifested as ischemic heart disease, chronic obstructive pulmonary disease, and chronic alcoholism was recorded.

## 4. Discussion

Unfortunately, we cannot compare our results with similar ones that were provided in the Russian Federation with the use of CCI in TB patients, which is due to the comorbidity status of TB treatment efficacy. This is the first attempt to analyze the long-term results of TB treatment efficacy in MDR and XDR TB patients. To date, the treatment of drug-resistant tuberculosis remains an important issue that has not been solved yet.

In recent years, drugs such as bedaquiline, delamanid, linezolid, clofazimine, and moxifloxacin have been used in treatment regimens for MDR/XDR tuberculosis treatment [26,27,28]. The analysis of adverse events showed that the fluoroquinolones clofazimine and bedaquiline had the lowest incidence of adverse events leading to definitive drug withdrawal. In contrast, the injectable drugs aminosalicylic acid and linezolid had the highest rate of adverse events. Colleagues rightly emphasize the need for careful monitoring of adverse events in patients with multidrug-resistant tuberculosis [28,29]. Concomitant cardiovascular pathology can markedly limit the application of bedaquiline, including its combination with moxifloxacin [19,30].

It is recognized that antituberculosis drugs exert hepatotoxic effects. Introducing new drug regimens in drug-resistant (DR) tuberculosis patients influences the level of therapy-related adverse events dramatically and affects therapeutic effectiveness in this patient cohort [4,9,31]. At the same time, no recommendations regarding the use of antihepatotoxicity drugs in tuberculosis patients, including those with MDR-TB and XDR-TB, have been proposed [14,17,32].

Comorbidity, being a concomitant pathology, differs profoundly from the underlying disease. However, it can influence patient management, drug administration, and TB treatment outcomes [32]. Severe manifestations of comorbid pathology can determine the prognosis of the underlying disease and its subsequent course, which is also true for tuberculosis [23]. According to the data of conducted studies, the level of comorbid pathology in TB patients is significantly higher due to socioeconomic inequality [6,32,33].

Patients with drug-resistant tuberculosis are characterized by alcohol, drug, and nicotine abuse that profoundly affects the disease course and mortality [7,34,35]. Such characteristics determine the patient’s social profile and affect adherence to TB treatment [7,8]. Compared with diabetes mellitus, alcoholism has been shown to have a greater impact on poor TB treatment efficacy and higher mortality rates [6]. At the same time, tobacco smoking is accompanied by the development of hypoproteinemia, which also accounts for the intensity of the tuberculosis course and affects the transmission of tuberculosis infection [35].

Diabetes mellitus and lack of hyperglycemic control are known to affect the course of tuberculosis infection [11,36,37]. However, no assessment of metabolic syndrome severity disorders has been published so far [38]. Diabetic patients are at high risk of developing active tuberculosis, requiring additional examination in the case of latent tuberculosis infection [39]. At the same time, no sufficient data on diabetes mellitus rates in MDR-TB and XDR-TB patients were reported, which is confirmed by our study data. A high percentage of gastrointestinal diseases and viral liver diseases and no diabetes mellitus were presented in MDR-TB patients upon an TB treatment failure course [10,11].

Hepatotoxicity is defined as damage associated with impaired liver function caused by drug exposure. The clinical picture of liver damage is presented as hepatocellular lesions coupled to a predominant increase in baseline alanine aminotransferase (ALT) levels, cholestatic signs related to elevated serum alkaline phosphatase concentration, or mixed signs combining rises in both enzyme levels [4]. Regular liver function monitoring is necessary to ensure patient safety during TB treatment [40]. Meta-analysis data revealed that liver function monitoring is required in tuberculosis patients receiving TB treatment [41,42].

The development of tissue hypoxia plays a crucial role in the pathogenesis of hepatocyte damage in chronic liver lesions resulting in impaired mitochondrial function [18,43]. Moreover, it is acknowledged that chemotherapy-related toxicity frequently results in morbidity and mortality in cancer patients and often results in complications in the medium term [44]. Data from previous studies revealed that biochemical parameters were improved along with a lowered hepatocyte necrobiosis level and decreased fatty dystrophy solely observed at the periphery of the lobules and within the inter-lobule space [45,46,47]. However, the administration of hepatoprotective and any other supportive treatment should be justified.

Some studies have attempted to identify an indicator of comorbidity severity and mortality risk [48,49]. The Charlson Comorbidity Index (CCI) was modified and improved based on pathology analysis [29,49]. It was shown that it allowed predicting long-term and in-hospital mortality rates in risk groups including patients in intensive care unit, oncologic pathology patients, and severe trauma patients. However, CCI has not been applied in tuberculosis patients, especially those with multidrug-resistant and extensively drug-resistant TB.

At present, no clear recommendations for the assessment of comorbid pathology in tuberculosis patients necessary to determine the management of patients with drug-resistant pathogens coupled with improved treatment outcomes have been proposed.

Such a strategy may not only improve therapeutic effectiveness in drug-resistant TB patients and lower the risk of adverse events, but also prevent patient treatment discontinuation. The introduction of new anti-TB drugs such as bedaquiline and delamanid decreased adverse reaction rates and increased patient adherence to treatment [50,51,52,53]. Obviously, XDR-TB patients comprise a patient cohort for which it is most difficult to select a therapeutic regimen. In this regard, even when bedaquiline was used in combination with other drugs, therapeutic effectiveness barely reached 71.9%, compared to 89.9% in MDR-TB patients [9,12].

Some studies revealed that the introduction of Bq into a treatment regimen in patients with CCI did not significantly change the therapeutic outcome with regard to the comparison group (40.9% and 34.7%, respectively, corresponding to 3–4 to 5–6 points) [54]. The data obtained suggest that regardless of comorbidity level, new drugs should be administered in treatment regimens for MDR and XDR TB patients. Hence, they imply the need for the introduction of novel therapeutic regimens in this patient cohort. In connection with this, revealing comorbidities and their correction at the earliest stages may contribute to further improving treatment effectiveness.

The findings of this study will potentiate further research on comorbid pathology. The growth of comorbid pathology, including in tuberculosis patients, requires the development of clear criteria for assessing the severity of comorbid pathology with a validated questionnaire. Currently, it is obvious that the emergence of new anti-TB drugs and their introduction are not as fast as they should be. This problem exists not only in the treatment of tuberculosis patients but also in the treatment of other infectious diseases. In this regard, the assessment of the severity of the comorbid status of tuberculosis patients can play a key role in the selection and prescription of a therapy scheme with the inclusion of new anti-TB drugs. Such an approach will help to avoid the development of adverse events, withdrawal of therapy, and treatment discontinuation. This is an important and primary element in the development of drug resistance of the pathogen to new drugs and TB treatment schemes. Preventive measures to counter the development of adverse events, especially in patients with comorbidities, may be cost-effective [55,56]. As a result, it will be important to lower the transmission of drug-resistant tuberculosis, which is of strategic importance in the context of newly emerging infectious agents.

## 5. Limitations of the Study

Obviously, we did not take into account the results of treatment of the most severe category of patients with tuberculosis combined with HIV infection. This should be the subject of a separate study. We also understand that the use of a method that is not validated for tuberculosis patients may have certain errors in the results. However, the data obtained are a starting point for initiating further studies to prevent the growth of drug-resistant tuberculosis. Additionally, we should keep in mind that by 2023, the criteria for evaluating tuberculosis with XDR-TB as the causative agent have changed. Therefore, it is necessary to conduct a study taking into account the new criteria. However, the data obtained on patients with XDR-TB can be used in the ways detailed in the following section.

## 6. Conclusions

The Charlson Comorbidity Index (CCI) can be a useful tool for objectively quantifying comorbid conditions in patients with XDR-TB. A higher CCI score indicates more comorbidities and a higher mortality risk. Assessing comorbidities is important in XDR-TB patients, as comorbid conditions can impact treatment tolerance, toxicity, and outcomes. Performing the CCI assessment can help identify patients who may require more intensive monitoring or adjusted treatment regimens. Appropriate management of comorbidities is an important part of the overall care for XDR-TB patients. Optimizing comorbid conditions may improve a patient’s ability to tolerate XDR-TB treatment and reduce complications. The calculation of the CCI before initiation of an XDR-TB regimen can aid clinical decision making regarding treatment approach, additional supportive care needs, and prognosis. It provides an objective metric for risk stratification. The regular reassessment of the CCI during treatment can also be used to monitor whether comorbidities are being adequately managed and controlled. In summary, assessing comorbidity burden with a tool like the CCI is a recommended part of the clinical evaluation and care planning for patients with XDR-TB. It can guide the management of accompanying conditions to optimize TB treatment outcomes.

## Figures and Tables

**Table 1:** Characteristics of patients with pulmonary drug-resistant tuberculosis.

Characteristics of Patients	Patients with Tuberculosis (n = 307)
Males (n (%))	218 (71.1)
Females (n (%))	89 (28.9)
Mean age (M ± m)	40.8 ± 11.2
Infiltrative lung TB (n (%))	148 (48.3)
Fibrotic cavernous lung TB (n (%))	95 (30.9)
Disseminated lung TB (n (%))	64 (20.8)
Comorbid pathology (n (%))	230 (74.9)
Treatment duration (years) (M ± m)	4.3 ± 2.5
Chronic alcoholism (n (%))	34 (11.1)
Smoking (n (%))	112 (36.5)

**Table 2:** Characteristics of patients.

Group of Patients	Group I—MDR-TB (n = 157)	Group II—XDR-TB(n = 150)	95% Cl	*p*, χ^2^
	n/%			
Length of therapy	3.1 ± 1.3 years	3.5 ± 1.5 years		
Previous anti-TB treatment	37 (23.6)	98 (65.3)	0.099; 0.269	*p* < 0.05; 0.54
Comorbidity	89 (58.2)	139 (92.6)	0.052; 0.207	*p* < 0.05; 0.53

**Table 3:** Treatment Outcome data in pulmonary MDR-TB and XDR-TB patients.

Group of Patients	Treatment Success (Cured + Treatment Completed)	Treatment Failure	Lost to Follow-Up	Death
	(n (%))			
Group I—MDR-TB (n = 157)	105 * (66.8)	45 (28.7)	6 (3.8)	0
Group II—XDR-TB (n = 150)	61 (40.7)	57 (38.0)	25 * (16.7)	7 * (4.6)

Notes: * *p* ˂ 0.01—data compared between Group I and Group II.

**Table 4:** Charlson Comorbidity Index in pulmonary MDR-TB and XDR-TB patients.

Patients with Comorbidities	Score (n, %)			
	0	1–2	3–4	5–6
Group I—MDR-TB (n = 89)	37 * (41.6)	30 (33.7)	22 (24.7)	0
Group II—XDR-TB (n = 139)	29 (20.9)	34 (24.5)	61 * (43.9)	15 * (10.7)

Notes: * *p* < 0.05—comparison of inter-group data.

**Table 5:** Comorbidity score in pulmonary MDR-TB and XDR-TB patients based on the effectiveness of therapy course.

Comparison Groups	Treatment Success (Cured + Treatment Completed) (n/%)			Treatment Failure (n/%)		
	1–2	3–4	5–6	1–2	3–4	5–6
	Score			Score		
Group I—MDR-TB (n = 89)	20 (38.5)	8 (15.4)	0	10 * (19.2)	14 (26.9)	0
Group II—XDR-TB (n = 139)	32 (29.1)	25 (22.7)	1 (0.9)	5 (4.5)	36 (32.7)	11 ** (10.0)

Notes: * *p* < 0.05—comparison of inter-group data; ** *p* ˂ 0.01—comparison of inter-group data.

**Table 6:** Statistically analyzed impact of comorbidity on TB treatment success.

Analyzed Relation	OR (95% CI)	*p*-Value
Comorbidity (CCI > 0) affects treatment success in entire sample	0.33 (0.16–0.67)	0.0019 **
Comorbidity (CCI > 0) affects treatment success in MDR-TB patients	0.3 (0.11–0.84)	0.018 *
High comorbidity index (CCI > 2) affects treatment success in XDR-TB patients	0.35 (0.13–0.95)	0.038 *
Effectiveness of TB treatment tending to decrease along with rise in comorbidity index in XDR-TB patients	Unapplicable	0.033 *

Notes: * *p* < 0.05—comparison of inter-group data; ** *p* ˂ 0.01—comparison of inter-group data.

**Table 7:** Concomitant pathology in XDR-TB patients with treatment failure.

Patients with TB Treatment Failure	Comorbidity Pathology (n (%))			
	CVD	COPD	PGIT and Viral Hepatitis	Chronic Alcoholism
XDR-TB (n = 42)	3 (7.1)	2(4.7)	32 (76.2) *	3 (9.1)
χ^2^	0.3	0.3	18.42	1.8
*p*	*p* > 0.05	*p* > 0.05	*p* < 0.001	*p* > 0.05

Notes: * *p*—significant difference for comparison of within-group parameter; CVD—cardiovascular disease; PGIT—pathology of the gastrointestinal tract; COPD—chronic obstructive bronchitis.

## Data Availability

Data are contained within the article.
